# The mediating role of social support in the relationship between alexithymia and anxiety in older women with breast cancer

**DOI:** 10.1192/j.eurpsy.2026.11557

**Published:** 2026-07-31

**Authors:** C. A. Bredicean, C. Oprean, S. Ursoniu, Z. Popovici, R. Homeag, G. Covaci, C. Giurgi-Oncu

**Affiliations:** 1 Neurosciences, NeuroPsy-Cog; 2 ANAPATMOL Research Center; 3 Functional Sciences, Victor Babes University of Medicine and Pharmacy, Timisoara; 4 Mental Health Center, Arad County Emergency Hospital, Arad; 5 Student, Victor Babes University of Medicine and Pharmacy; 6 Psychiatry, “Dr. Victor Popescu” Emergency Military Clinical Hospital, Timisoara, Romania

## Abstract

**Introduction:**

Older women with breast cancer are particularly vulnerable to emotional distress. **Alexithymia**, defined as difficulty recognizing and expressing emotions, is one of the factors that may influence the course of the disease. It is also frequently associated with anxiety. The relationship between alexithymia and anxiety is mediated by several factors, including social support.

**Objectives:**

Evaluating the role of **social support** as a mediator in the relationship between **alexithymia** and **anxiety** in elderly women with breast cancer.

**Methods:**

The study was conducted on women (N-83) over 65 years of age who were diagnosed with breast cancer. Their evaluation was done using a cross-sectional design that included: alexithymia (TAS-20 Toronto Alexithymia Scale), anxiety (DASS – Anxiety subscale), and social support (assessed through a standardized questionnaire). Statistical analyses included Pearson correlations and the mediation model based on regression, according to the Baron & Kenny method.

**Results:**

The direct correlation between alexithymia and anxiety was positive, but not statistically significant (r = 0.22, p = 0.051). Mediation analysis revealed a significant indirect effect: alexithymia negatively predicted social support (*a* = 0.28, *p* < 0.001). Lower social support scores predicted higher levels of anxiety (*b* = 0.32, *p* = 0.025). The direct effect of alexithymia on anxiety became insignificant after social support was introduced into the model (*c’* = 0.10, *p* = 0.34). Our results indicated that social support fully mediates the relationship between alexithymia and anxiety in older women.

**Conclusions:**

In elderly women diagnosed with breast cancer, **social support has an essential protective role**, reducing anxiety even in the presence of high alexithymia scores. Interventions focused on **increasing support networks and psychosocial support** may contribute to improving the quality of life in this vulnerable population. Adequate social support may also improve the prognosis and evolution of the oncological disease.

**Disclosure of Interest:**

None Declared

